# Explaining Online Information Seeking Behaviors in People With Different Health Statuses: German Representative Cross-sectional Survey

**DOI:** 10.2196/25963

**Published:** 2021-12-10

**Authors:** Elena Link, Eva Baumann, Christoph Klimmt

**Affiliations:** 1 Department of Journalism and Communication Research Hanover University of Music, Drama and Media Hanover Germany

**Keywords:** online health information seeking behavior, Planned Risk Information Seeking Model, health status, theory building, personal survey

## Abstract

**Background:**

Worldwide, the internet is an increasingly important channel for health information. Many theories have been applied in research on online health information seeking behaviors (HISBs), with each model integrating a different set of predictors; thus, a common understanding of the predictors of (online) HISB is still missing. Another shortcoming of the theories explaining (online) HISB is that most existing models, so far, focus on very specific health contexts such as cancer. Therefore, the assumptions of the Planned Risk Information Seeking Model (PRISM) as the latest integrative model are applied to study online HISB, because this model identifies the general cognitive and sociopsychological factors that explain health information seeking intention. We shift away from single diseases and explore cross-thematic patterns of online HISB intention and compare predictors concerning different health statuses as it can be assumed that groups of people perceiving themselves as ill or healthy will differ concerning their drivers of online HISB. Considering the specifics of online HISB and variation in individual context factors is key for the development of generalizable theories.

**Objective:**

The objective of our study was to contribute to the development of the concept of online HISB in 2 areas. First, this study aimed to explore individual-level predictors of individuals’ online HISB intention by applying the postulates of PRISM. Second, we compared relevant predictors of online HISB in groups of people with different health statuses to identify cross-thematic central patterns of online HISB.

**Methods:**

Data from a representative sample of German internet users (n=822) served to explain online HISB intentions and influencing patterns in different groups of people. The applicability of the PRISM to online HISB intention was tested by structural equation modeling and multigroup comparison.

**Results:**

Our results revealed PRISM to be an effective framework for explaining online HISB intention. For online HISB, attitudes toward seeking health information online provided the most important explanatory power followed by risk perceptions and affective risk responses. The multigroup comparison revealed differences both regarding the explanatory power of the model and the relevance of predictors of online HISB. The online HISB intention could be better explained for people facing a health threat, suggesting that the predictors adopted from PRISM were more suitable to explain a problem-driven type of information-seeking behavior.

**Conclusions:**

Our findings indicate that attitudes toward seeking health information online and risk perceptions are of central importance for online HISB across different health-conditional contexts. Predictors such as self-efficacy and perceived knowledge insufficiency play a context-dependent role—they are more influential when individuals are facing health threats and the search for health information is of higher personal relevance and urgency. These findings can be understood as the first step to develop a generalized theory of online HISB.

## Introduction

### Relevance of Focusing on Online Health Information Seeking Behavior

The internet occupies an increasingly important site for health information in many regions of the world [1,2]. For instance, in the United States, 80.2% of the population search for health-related information via the internet [2]. In Germany, using the internet to seek health-related information is also of increasing relevance. A national survey showed that 72% of the population use the internet to seek information on health issues [3]. The internet provides an active, problem-oriented opportunity to find a high volume and variety of online health information available virtually anywhere and anytime. That information can be used to guide individuals’ health-related decisions, help to cope with uncertainties, and find strategies for living with health threats. Online health information seeking behaviors (HISBs) give individuals more control over their health care and greater knowledge about their condition [4,5]. Doing so, online HISB is a “key facilitator for promotion, maintaining, and returning to health” [6]. Research on why people seek health information online can identify which specific groups can be reached using online sources for health interventions [7]. Therefore, it is important to learn more about the online search for health information and to understand individuals’ choices that determine their online HISB.

A wide range of theories has been applied or used as guidance to examine online HISB, with each model integrating a different set of predictors [5,6,8-12]. Thus, a common understanding of predictors of online HISB is still missing [13]. To learn more about the dominant predictors of online HISB, this study aims to explore individual-level influencing factors that lead people to seek health information online. Further, we adopted the Planned Risk Information Seeking Model (PRISM; [14]) as the latest integrative model for the online domain and test its applicability. Another shortcoming of the theories explaining HISB and online HISB that our study aims to address is that most existing models, so far, focus on very specific health contexts such as cancer or diabetes [9,15-18]. Contrary to this focus, a trend in information seeking research follows Case’s [19] call to shift away from single diseases [14,20,21]. In response to this requirement, we aim to explore cross-thematic patterns of online HISB. We use health status as a comparative dimension because it can be assumed that groups of people perceiving themselves as ill or healthy will differ concerning the personal relevance of online HISB and its drivers [21-23]. Considering variations in individual context factors is the key for the development of generalizable theories because it helps to identify both context-specific determinants and cross-contextually important predictors that would emerge as the core of online HISB [6].

To sum up, the objective of our study was to contribute to the theoretical development of the concept of online HISB concerning 2 areas. First, we aimed to apply the PRISM to online HISB to analyze predictors of online HISB in general. Second, we compared the relevant predictors of online HISB in groups of people with different health statuses to identify cross-thematic central patterns of online HISB.

### Approaches and Challenges of Health Information Seeking in the Online Domain

HISB is a complex, often multistage, process that can be conceptualized by its triggers, channel selection, search strategy, types of information sought, and outcomes [24]. Johnson and Case [6] describe the selection of a channel as the most basic decision individuals can make regarding information seeking. According to the assumptions of a goal-directed selection of information channels, the specific characteristics of a channel influence the intention to turn to it [25-29]. Even though the combined use of multiple channels for information seeking is prevalent [14,19,30], we argue that a comprehensive understanding of the different channel-specific processes of HISB is needed. Therefore, we consider internet-specific capabilities, attitudes, and norms as access points for further theory development [8,31]. Compared with other channels, the internet offers permanent, 24-hour access to extensive, multifaceted, in-depth, and latest health information. Furthermore, the active and goal-oriented options to search for health information online allow a high degree of customizability [11,12,32]. It is also suitable to seek sensitive information in contexts where anonymity is of high relevance [33]. Beyond channel characteristics, explanations for why people go online have been found in specific predictors. Powell and colleagues [34] described the characteristics of online HISB identifying certain motivations, such as the desire for reassurance, a second opinion, and better understanding of information. However, information with varying quality raises the importance of certain abilities and perceived internet self-efficacy to find reliable and accurate health information increases [12,32,35]. In sum, existing theory and research on HISB should be extended by taking channel characteristics of the internet into account and modeling online HISB.

### Modeling Online HISBs

The high importance of the internet for HISB has motivated a large number of studies, most of which aim to describe and explain internet use for health-related purposes [36]. Because of the wide range of theories and predictors used to examine online HISB [8,9], it remains unclear which predictors are the dominant or universal ones for online HISB. A specific model of online HISB is missing; thus, in our study, a well-established model of HISB serves as a foundation for modeling online HISB to take past theoretical progress in general HISB into account to allow progress. The assumptions of the PRISM [14] are adopted to study online HISB because this model identifies the general cognitive and sociopsychological factors that motivate HISB. Variables considered in PRISM were already applied to examine online HISB [13], but the whole model was not examined regarding online HISB so far.

The PRISM was developed with a general health reference and aimed at explaining information seeking intention in light of individual-level factors across different risk- and health-related issues that are valid for multichannel HISB [14]. PRISM is an integrated model postulating the importance of 7 distal and proximal factors applied from models such as the Theory of Planned Behavior [37], the Risk Information Seeking and Processing Model (RISP; [22,23]), and the Comprehensive Model of Information Seeking [8,26]. It is often viewed as an expanded iteration of the RISP that has incorporated additional models to explain general information seeking intentions as an outcome [30]. Because of this comprehensiveness, we deemed the PRISM particularly suitable for our approach.

Based on the Theory of Planned Behavior, the PRISM posits that the intention to seek information is the result of attitudes toward information seeking, subjective seeking-related norms, as well as perceived seeking control. Attitudes toward information seeking capture an individuals’ evaluation of the information-seeking behavior. Subjective norms consist of perceived expectations or social pressure of one’s social surroundings to seek information (injunctive norms) as well as seeking behaviors perceived in one’s social surroundings (descriptive norms). The perceived behavioral control is understood as an individual's ability to seek information [14]. Further, adopted from the RISP, the PRISM includes an individual’s perception of his/her state of knowledge and knowledge insufficiency as well as health-related risk perception and affective risk response [21-23] as factors that influence the intention to search for information. Knowledge and knowledge insufficiency depict the assumption that the desired level of confidence in an individuals’ knowledge motivates information seeking [14,21]. Knowledge insufficiency is the gap between what an individual knows and what he or she needs to know to feel confident [23,30]. Risk perception refers to the susceptibility and severity of risks to one’s health, whereas affective risk response describes a negative affective reaction induced by the health risk [14,21]. All predictors are postulated to be positively related to the HISB intention.

Kahlor [14] postulated that the predictors integrated into the PRISM are valid across channels, but the claim has never been empirically tested and the individual explanatory power of the predictors remains uncertain. Particularly, some past studies including review and meta-analysis indicated that the power of the predictors of online HISB differs from that of offline sources [8,31,38,39]. Therefore, Wang and coauthors [13] concluded that there is a need to refine the PRISM for online HISB.

Concerning attitudes toward seeking and subjective norms, a meta-analytical review [13] showed that the relation between both predictors and online HISB is understudied. About the attitude, recent research postulated a positive association between attitudes toward online HISB and online HISB intention [13], suggesting that the postulates of the PRISM apply to the online context. Comparing source usage regarding the association with norms, the first findings are mixed. Although studies support that subjective norms are a critical antecedent of online HISB [40], others found that normative influences of family and friends motivate the choice of interpersonal and mass media channels but not the internet [39]. Accordingly, the results at least call into question whether norms are among the strongest predictors of online HISB, as presumed for HISB [21].

Besides, recent studies stressed that particular (perceived) abilities gain importance for health-related internet use compared with other sources [13,31]. Instead of perceived seeking control that is considered in the PRISM [14], perceived self-efficacy is often theorized as a factor influencing internet use [8,13,35]. The terms are sometimes used synonymously, or self-efficacy is understood as a dimension of seeking control [20,41]. However, although perceived seeking control includes internal abilities and external capabilities such as the accessibility of information, self-efficacy is predominantly based on internal control factors referring to beliefs in one’s capabilities to execute a certain course of action such as online HISB [42]. Therefore, we categorize perceived self-efficacy as a part of perceived seeking control [43]. Because more than 90% of German residents possess access to the internet [44] and Germany is characterized by a high information and communication technology development index, external barriers to information acquisition are perceived as low, whereas own capabilities might be a relevant predictor of online HISB. In line with studies showing that self-efficacy is a well-confirmed predictor of internet use [35], we aim to focus on perceived internal control factors of those residents having access to the internet. Acquiring health information online is understood as a behavior that requires many unique capabilities from learning how to use the internet or search engines to gather health information, locating different perspectives or high-quality information, and evaluating the quality of health websites. The need for corresponding abilities underscores the importance of efficacy perceptions to perform online HISB, as self-efficacy is known to determine how much effort an individual is willing to invest to perform a certain course of action such as online HISB [35].

The multichannel comparison of Yang et al [39] highlighted that risk perception and affective risk responses as well as current knowledge and knowledge insufficiency had different impacts on HISB depending on the channel used. For online HISB, risk perception, current knowledge, and knowledge insufficiency were shown to be of low relevance for online HISB [39]. For negative affective risk responses, a weak but a significant positive association with online HISB was supported, whereas negative responses were not associated with turning to interpersonal or other media sources [39]. Given past findings that the internet often serves as a channel for a second opinion [11,35,45], prior use of other sources might result in higher perceived knowledge or higher awareness of knowledge insufficiency as predictors of online HISB and influence the risk perception and affective risk responses, which might influence the selection of the internet.

**Figure 1:**
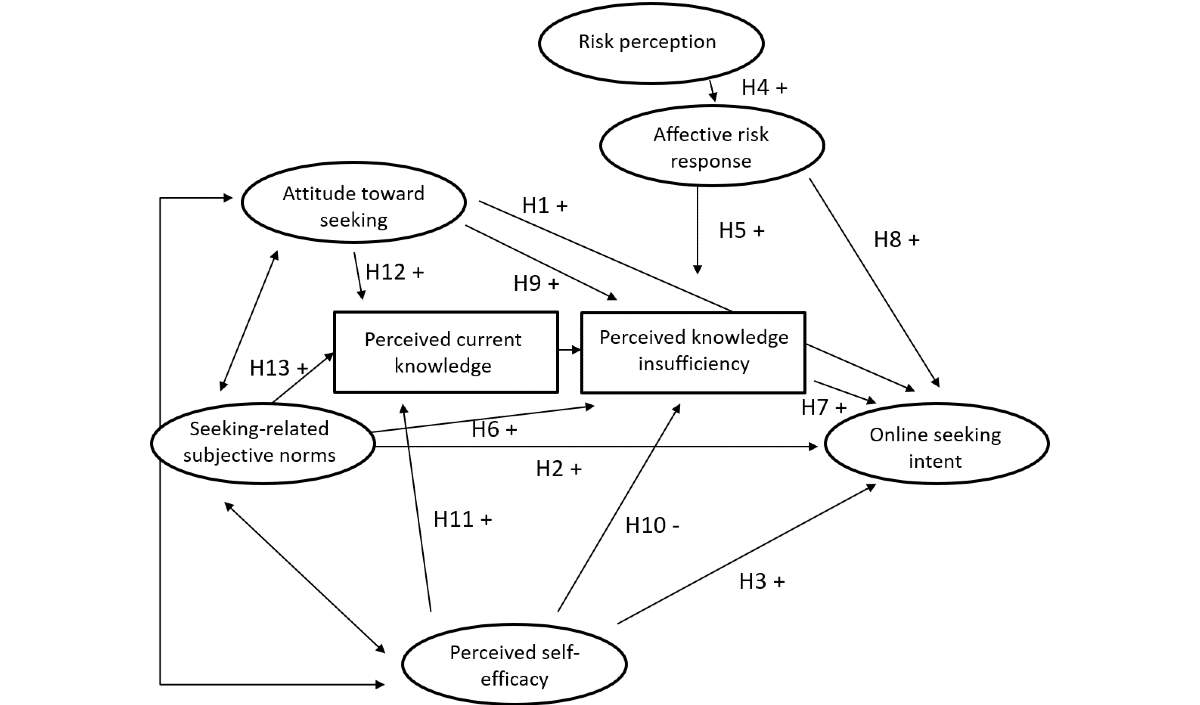
Predicted relationships of the Planned Risk Information Seeking Model applied to online health information seeking behavior. H: hypothesis.

Overall, these findings indicate that established factors of HISB such as social norms, informational self-efficacy, risk perception, affective risk response, and knowledge insufficiency are of relevance for online HISB and increase the intention to seek health information online. However, the dominance of the predictors might differ in comparison to HISB in offline channels. Therefore, it is indicated to test whether the general assumptions of the PRISM can be applied for explaining online HISB. Our first research question (RQ1) is as follows: *Can the PRISM be applied to explain online HISB intention?* The single hypotheses (H1 to H13) reflect the predicted relationships of PRISM, whereas the single predictors were transferred to online HISB. The single hypotheses are shown in Table 1 and illustrated in Figure 1.

### Health Status–Related Importance of Predictors of Online HISB

Aiming to explain online HISB beyond single disease contexts, we consider the personal relevance of health topics [39] distinguishing between routine (ie, driven by a general interest) and nonroutine information seeking (ie, triggered by a specific health challenge; [23,33,46]). This context factor of online HISB is considered by exploring differences in the importance of predictors of online HISB between a generally healthy population and people who are facing current health issues. Current research considering health-related context factors of online HISB mainly considered health status as a predictor of online HISB frequency but did not distinguish predictors of online HISB depending on different health-related contexts [1,4,9,10,42]. However, the PRISM and RISP have already been tested concerning different communities and different risks, that is, across differing health-related contexts [16,23,39,47-50]. Findings revealed the consistency of factor importance across different risks [23,51] but showed first indications of differences between populations with limited versus high personal relevance of a health topic [39]. More detailed findings of Yang et al [39] suggested that in the general population, higher perceived risks increased online HISB, whereas among patients with cancer, risk perception caused a decrease of online HISB. Furthermore, people’s sense of knowledge insufficiency varied based on health status, and normative beliefs had a stronger impact on online HISB in the general population than that among patients with cancer [39,51,52]. Further, the attitudes toward seeking information online can be determined by health status as they are associated with the salience of perceived channel characteristics [28] and the perceived utility of the internet [8]. As perceived health threats may reduce the self-ascribed ability to succeed in online HISB [1,53], it is conceivable to posit a context-dependent role of self-efficacy to use the internet for health-related purposes. Recent research has suggested that health status may result in different influence patterns of online HISB, which might contradict the cross-channel universality of the PRISM. To substantiate this assumption, a comparison of the empirical importance of predictors in groups of individuals with different health statuses is required. Thus, our second research question (RQ2) is as follows: *Do the direct and indirect predictors of online HISB intention differ between people who face a health threat and people who perceive themselves as healthy?*

## Methods

### Recruitment Procedure and Participants

We conducted a standardized personal survey with a representative sample of 1001 German individuals. The fieldwork was conducted by a German market research company using quota sampling intended to guarantee representative population data. Because we aimed to explain online HISB, our analysis included only people with internet access (822/1001, 82.1% of the respondents). The respondents were aged between 18 and 88 years (mean 47.08 [SD 16.29] years), and more than half of the participants were females (459/822, 55.8%). Regarding school education, the largest proportion of participants had a secondary school leaving certificate (371/822, 45.1%). Approximately 28.6% (235/822) of respondents had less than a secondary school leaving certificate, 14.8% (122/822) had graduated from high school, and 11.4% (94/822) had university degrees.

### Measures: Online Seeking Intention

Based on Kahlor’s study [14], 3 out of 5 items were applied to the internet that, on the one hand, express a different strength of intention (“I intend to look for health information on the internet in the near future;” “I will look for health information related to my personal health and risks to my health in the near future”) and, on the other hand, aim at intensifying the search for information in the future (“I intend to find more information about my health on the internet soon”). A global query that refers to the internet as a whole seemed justified, as recent studies [45,54] and our survey confirmed that search engines were central (mean 3.36 [SD 0.96]; frequency measured on a 5-point Likert-type scale ranging from 1 “never” 1 to 5 “very often”), while direct access to specific online communities (mean 1.68 [SD 0.95]) or health websites (mean 2.04 [SD 1.01]) was rarely practiced. Further, in the United States, 77% of online health seekers reported using a search engine to begin a search, whereas only 13% began at a site that is specialized in health information [54]. The 3 items were rated on a 5-point Likert-type scale ranging from strongly disagree (1) to strongly agree (5) (α=.920; mean 2.45 [SD 1.13]).

### Measures: Predictors

#### Attitudes Toward Online Information Seeking

We adopted the measurement from Kahlor [14] and instructed our participants to describe their attitudes toward online HISB with seven 5-point semantic differential pairs (eg, bad/good, unhelpful/helpful; α=.944; mean 3.29 [SD 0.90]).

#### Seeking-Related Social Norms

To measure injunctive and descriptive norms, we used single items adopted from Kahlor [14]: “My family and friends expect me to seek information on health-related topics and risks” (mean 2.75 [SD 1.15]; injunctive norm) and “People in my life whose opinions I value seek information on risks and health-related topics” (mean 3.37 [SD 0.98]; descriptive norm). Both items were evaluated on a 5-point Likert-type scale: strongly disagree (1) to strongly agree (5). Since injunctive and descriptive norms are theoretically conceptualized as 2 dimensions of social norms [52,55,56] and their correlation is mediocre (r=0.38; *P*=.01), we decided to treat them as distinct concepts.

#### Perceived Health-Related Internet Self-efficacy

Internet self-efficacy was assessed based on a scale of Eastin and LaRose [57], which was adapted and applied to online HISB by Rains [35]. The measure consists of 8 items describing different tasks (eg, evaluating the quality of health websites, finding high-quality health information) asking for respondents’ perceived abilities in using the internet to acquire health information. Participants’ applicability to the statements was measured on a 5-point Likert-type scale (α=.947; mean 3.51 [SD 0.97]).

#### Risk Perception and Affective Risk Response

Following Kahlor [14], we measured risk perception asking for the susceptibility (“How likely are you to become ill in the next year?”) and the severity of an illness (eg, “If you were to become ill in the next year, how serious do you think it would be?”). All items for risk perception were measured on a Likert-type scale ranging from 1 (not at all) to 10 (extremely; α=.863; mean 3.14 [SD 1.89]). As affective responses to perceived health risks, we assessed the extent to which the participants felt worried, scared, or uncertain (α=.963; mean 3.32 [SD 2.21]). The items that capture affective risk response were measured on a Likert-type scale ranging from 1 (not at all) to 10 (extremely).

#### Perceived Knowledge and Knowledge Insufficiency

In line with Kahlor [14], we assessed knowledge and knowledge insufficiency on scales from 0 to 100. First, we asked the respondents to rate their current state of knowledge about health-related topics (mean 57.69 [SD 23.01]). Second, they were asked to rate their needed level of knowledge to cope with health-related topics (mean 73.49 [SD 19.7]).

#### Health Status

Health status was measured in 2 different ways [58]. First, respondents were asked to rate their health status (mean 3.35 [SD 0.85]) on a 5-point scale from “poor” (1) to “excellent” (5). Second, we asked whether the participants perceived themselves as currently completely healthy, acutely slightly ill, acutely seriously ill, or chronically ill [58]. Additionally, we provided the option to refuse to answer this question. To have a sufficient group size for comparison, the answers “acutely slightly ill” (154/822, 18.7%) or “acutely seriously ill” (16/822, 1.9%) and “chronically ill” (59/822, 7.2%) were merged (see RQ2). For this purpose, 2 groups—people perceiving themselves as healthy (n=564) or ill (n=229)—were built (see Multimedia Appendix 1, Table S1). Of the 822 participants, 29 (3.5%) refused to answer the question about their health status and were not considered for the group comparison (see RQ2). To validate this measurement, the relationship between both measurements of health status was assessed showing a rather strong association (r=0.53; *P*=.01).

### Statistical Analysis

To answer our research questions and test our hypotheses, we used latent-variable structural equation modeling in R (version 3.4.2). We used two-step modeling to verify all measurement models before testing the structural model. The data fit of all measurement models was evaluated as satisfying (see Multimedia Appendix 1). In response to the second research question, we conducted a multigroup analysis to test the PRISM applied to online HISB intention in a group comparison for healthy and ill internet users. Therefore, measurement and structural invariance were evaluated. The results for measurement invariance are shown in Multimedia Appendix 1. The invariance appeared problematic but justifiable in single cases (see Multimedia Appendix 1). The structural invariance was determined by comparing the unconstrained and constrained model using χ² and fit statistics.

## Results

**Figure 2:**
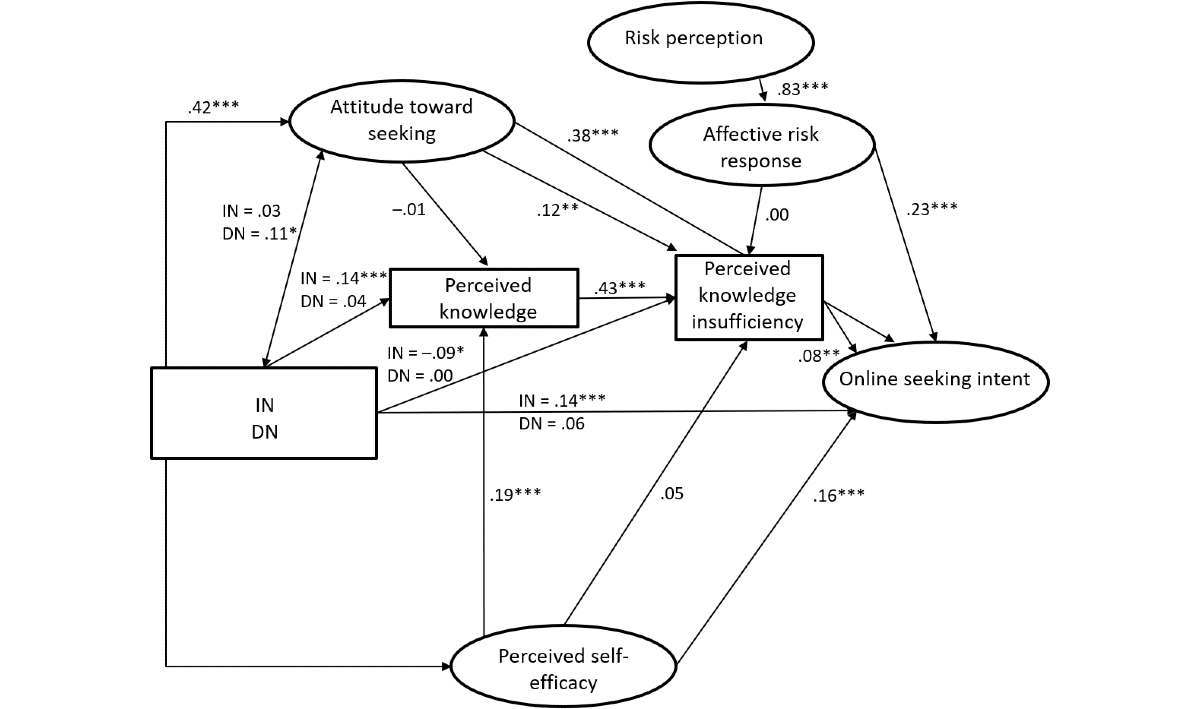
Planned Risk Information Seeking Model applied to online health information seeking behavior intention. The results of the hypotheses tests are shown as standardized beta coefficients and their significance. DN: descriptive norm; IN: injunctive norm. *P<.05, **P<.01, ***P<.001.

RQ1 asked if the PRISM predictors can be applied to explain online HISB. The model fit indices showed a satisfactory model fit (*χ*²_335_=846.5, *P*≤.001; comparative fit index [CFI]=0.962, root mean square error of approximation [RMSEA]=0.043, standardized root mean square residual [SRMR]=0.054; [59,60]). Since the other indices had very satisfactory levels, the significant *χ*²-test was attributed to the sample size [61,62]. The model accounted for 31.8% of the variance in online HISB intention. Besides, the model explained 21.9% of the variance in perceived knowledge insufficiency, 5.3% of the variance in perceived knowledge, and 68.5% of the variance in affective risk response. The results of the hypotheses tests (standardized beta coefficients and their significance) are reported in Table 1 and Figure 2. Overall, 7 of the 13 hypotheses were confirmed. Paths that could not be confirmed applied to online HISB were associated with the distinction between injunctive and descriptive norms. Only influences of injunctive norms were found. Injunctive norms were positively related to online HISB intention (see H2) and perceived knowledge (see H13) but negatively related to perceived knowledge insufficiency (see H6). Additional relationships that could not be supported for online HISB intention included the paths between affective risk response and perceived knowledge insufficiency (see H5), between perceived self-efficacy and perceived knowledge insufficiency (see H10), and between attitudes toward seeking and perceived knowledge (see H11). In contrast to previous studies focusing on HISB [14,47,50], the path between perceived knowledge insufficiency and online HISB intention was confirmed (see H7).

**Table 1:** Overview of the hypotheses and outcomes.

Proposed path (H: hypothesis)	Online PRISM^a^	Online PRISM (group: healthy)	Online PRISM (group: ill)
H1: Attitude toward seeking (online) will be positively related to online information–seeking intent.	Supported	Supported	Supported
H2: Seeking-related subjective/injunctive and descriptive norms will relate positively to online information–seeking intent.	IN^b^: Supported DN^c^: Not supported (not significant)	IN: Supported DN: Not supported (not significant)	IN: Not supported (not significant) DN: Supported
H3: Perceived seeking control/self-efficacy to search for information (online) will be positively related to online information–seeking intent.	Supported	Not supported (not significant)	Supported
H4: Risk perceptions will be positively related to affective risk response.	Supported	Supported	Supported
H5: Affective risk response will relate positively to perceived knowledge insufficiency.	Not supported (not significant)	Not supported (not significant)	Not supported (not significant)
H6: Seeking related subjective/injunctive and descriptive norms will relate positively to perceived knowledge insufficiency.	IN: Not supported (negatively related) DN: Not supported (not significant)	IN: Not supported (negatively related) DN: Not supported (not significant)	IN: Not supported (negatively related) DN: Not supported (not significant)
H7: Perceived knowledge insufficiency will relate positively to information seeking intent.	Supported	Not supported (not significant)	Supported
H8: Affective risk response will be positively related to (online) information–seeking intent.	Supported	Supported	Supported
H9: Attitude toward seeking (online) will relate positively to perceived knowledge insufficiency.	Supported	Not supported	Supported
H10: Perceived seeking control/self-efficacy will be negatively related to perceived knowledge insufficiency.	Not supported (not significant)	Not supported (not significant)	Not supported (not significant)
H11: Attitude toward seeking will be positively related to perceived knowledge.	Not supported (not significant)	Not supported (not significant)	Not supported (not significant)
H12: Perceived seeking control/self-efficacy will be positively related to perceived knowledge.	Supported	Supported	Supported
H13: Seeking-related subjective/injunctive and descriptive norms will be positively related to perceived knowledge.	IN: Supported DN: Not supported (not significant)	IN: Supported DN: Not supported (not significant)	IN: not supported (not significant) DN: Not supported (not significant)

^a^PRISM: Planned Risk Information Seeking Model.

^b^IN: injunctive norm.

^c^DN: descriptive norm.

RQ2 asked for differences of predictors of online HISB between groups of people perceiving themselves as healthy or ill. Comparing the unconstrained and constrained model showed that both models fit the data fairly well (unconstrained: *χ*²_686_=1295.0, *P*≤.001; CFI=0.958; RMSEA=0.050; SRMR=0.061; constrained: *χ*²_726_=1295.0, *P*≤.001; CFI=0.949; RMSEA=0.051; SRMR=0.065), but the *χ²* difference test indicated that the models are not equivalent (Δ*χ²*=101.1, Δdf=40; *P*≤.001). The unconstrained model was superior, implying that path coefficients vary among groups. The comparison of the power of the model for healthy and ill people showed that the model accounted for a higher level of variance for ill people with regard to online HISB intention (healthy people: R^2^=0.273 vs ill people: R^2^=0.437; ΔR^2^=0.164), the affective response to perceived risks (healthy people: R^2^=0.550 vs ill people: R^2^=0.693; ΔR^2^=0.143), and perceived risks (healthy people: R^2^=0.058 vs ill people: R^2^=0.074; ΔR^2^=0.016). In turn, the model explains more variance in perceived knowledge insufficiency for healthy people (R^2^=0.229) in comparison with ill people (R^2^=0.193; ΔR^2^=0.036).

**Figure 3:**
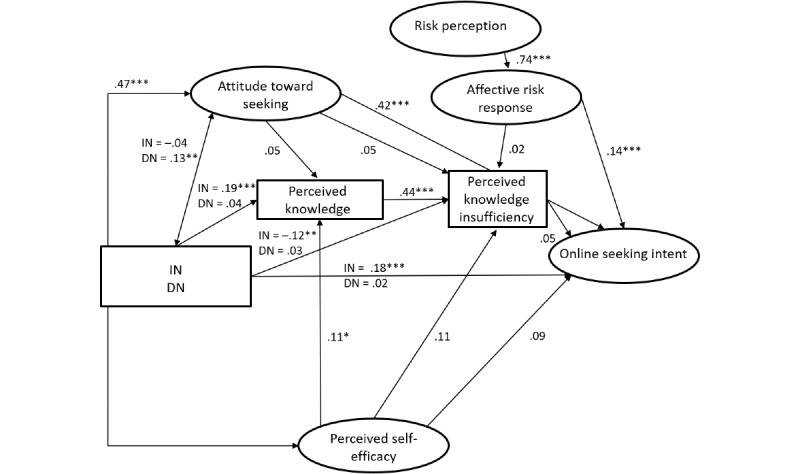
Online Planned Risk Information Seeking Model comparing healthy and ill people (group of healthy individuals). The results of the tests are shown as standardized beta coefficients and their significance. DN: descriptive norm; IN: inductive norm. *P<.05, **P<.01, ***P<.001.

**Figure 4:**
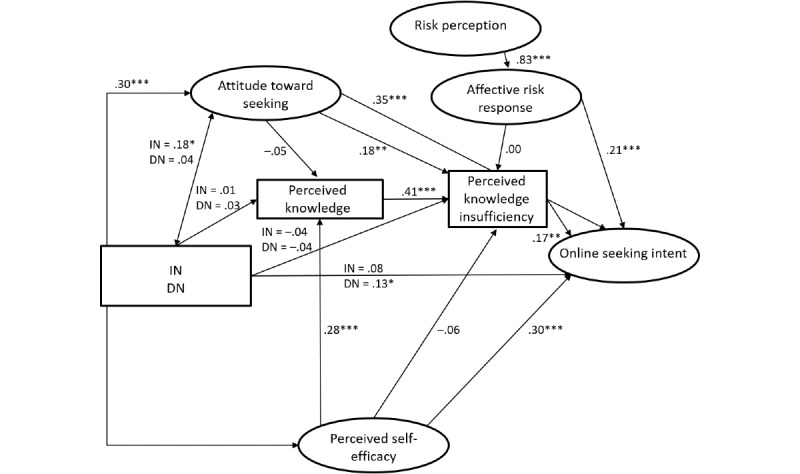
Online Planned Risk Information Seeking Model comparing healthy and ill people (group of ill individuals). The results of the tests are shown as standardized beta coefficients and their significance. DN: descriptive norm; IN: inductive norm. *P<.05, **P<.01, ***P<.001.

Looking at single paths (see Figure 3 and Figure 4), we found different influences from social norms (see H2 and H13). While in the healthy group, injunctive norms had weak to mediocre but significant effects on seeking intention (healthy: β=.18; *P*<.001; ill: β=.08; *P=*.20), on perceived level of knowledge (healthy: β=.19; *P*<.001; ill: β=.01; *P*=.91), and on perceived knowledge insufficiency (healthy: β=–.12; *P*=.01; ill: β=–.04; *P*=.55), these paths were not significant or weaker in the group of ill people (Figure 4).

For the group of ill people, the intention to seek information was significantly influenced by descriptive norms (healthy: β=.02; *P*=.61; ill: β=.13; *P=*.047) rather than injunctive norms. Furthermore, perceived internet self-efficacy had a significant and stronger influence on the online HISB intention (see H3; β=.09; *P=*.07 vs ill: β=.30; *P*<.001) and the perceived level of knowledge among ill people (see H12; healthy: β=.11; *P*=.049 vs ill: β=.28; *P*<.001). The association between self-efficacy and attitudes toward seeking was stronger in the group of healthy people compared with people facing health threats (healthy: β=.47; *P*<.001; ill: β=.30; *P*<.001). Other differences related to the relationships between perceived knowledge insufficiency and health information seeking intention (see H7; healthy: β=.05; *P*=.19; ill: β=.17; *P*=.01) and between the attitudes toward seeking information online and knowledge insufficiency (see H9; healthy: β=.05; *P*=.31; ill: β=.18; *P*=.01). In both cases, only the path for the group of people perceiving themselves as ill was significant.

## Discussion

### Specifics of the Predictors of Online HISB Intention

As the internet is a frequently used channel for health-related purposes, we applied the PRISM [14] to the internet to provide a theoretically sound analysis of predictors of online HISB intention and refine the PRISM for the online context (RQ1). Our results reveal PRISM to be an effective framework for explaining online HISB intention. For online HISB, attitudes toward seeking health information online provide the most important explanatory power followed by risk perceptions and affective risk responses. These factors are identified as most essential for online HISB intention and can be understood as the cross-contextual core or dominant predictors of online HISB [6,13]. Further, perceived health-related internet self-efficacy, injunctive norms, and perceived knowledge insufficiency influence a higher online HISB intention. However, they seem to be of secondary importance to explain the intention to turn to the internet. For self-efficacy, our findings do not confirm the postulated higher importance for online HISB [31]. This may be attributed to the fact that our respondents perceive internet use for health-related purposes as less challenging than assumed. As the analyzed sample consists of internet users only, it would be a valuable supplement to compare users with nonusers. However, this was not the aim of this study but should be considered in the future. Regarding the rather weak impact of social norms, our findings are in line with the results of Yang et al [39]. Social norms seem to be less important for online HISB in comparison with HISB in general [14,16]. This is particularly evident for descriptive norms, which have neither an effect on the intention to seek information nor on perceived knowledge insufficiency and the current state of knowledge. The rather weak effect of perceived knowledge insufficiency should be assessed against the background of previous studies that found no relation between knowledge insufficiency and seeking intention [14,50]. This raises the question about the importance of perceived knowledge as a relevant predictor of seeking intention [21]. Our results indicate that the PRISM is a fruitful base to explain online HISB intentions and demonstrate that the process of online HISB rests partially on influencing factors other than offline HISB [21,39]. Thus, more research comparing online HISB with HISB in other channels is needed to determine the differences between channels and to develop a robust generalizable theory of online HISB.

### Impact of Health Status on Online HISB

RQ2 addressed the difference between predictors determining online HISB intentions for groups of people perceiving themselves as healthy or ill. The multigroup comparison revealed differences both regarding the explanatory power of the model and the relevance of different influencing factors of online HISB. In general, the causal assumptions were confirmed with greater effect sizes in the subsample of ill people than that in the healthy subsample. Accordingly, the online HISB intention can be better explained for people facing a health threat, suggesting that the influencing factors adopted from PRISM are more suitable to explain a problem-driven type of information-seeking behavior [33,46,63]. These findings call for improving the theoretical understanding of online HISB intentions by healthy people who do not feel immediate pressure to acquire health-related knowledge even though they might perceive their knowledge as insufficient. At the same time, these results indicate that the internet is a particularly important source for ill people [3,45,64], which is important to consider in efforts of distributing health information.

Referring to the single predictors, attitudes toward seeking and risk perception are identified as important in both groups. Although attitudes toward seeking have a stronger influence in the group of healthy people, risk perception and affective risk response show a stronger association for people facing health threats. This contrasts with the findings of Yang et al [39], who identified a positive effect of risk perception on internet use for healthy people and a negative effect for patients with cancer. The severity of a disease could be responsible for this difference. It should be pointed out that the group of people facing a health threat surveyed in this study covers very different health statuses; hence, it is impossible to address differences related to diseases with different degrees of severity and susceptibility. Specific influencing patterns are plausible, and non-HISB coping strategies such as information avoidance are more likely in the case of highly severe diseases such as cancer [65].

Beyond the cross-contextual factors of online HISB, social norms, internet self-efficacy, and perceived knowledge insufficiency are observed as context-specific influencing factors of online HISB intentions. The results regarding the role of social norms are in line with the findings of Yang et al [39] and complement them. They confirm that injunctive norms are a more important predictor for healthy than for ill people. Besides, our data show a higher relevance of descriptive norms for people perceiving health threats. Thus, the individual adherence to injunctive and descriptive norms indicates that healthy people are more strongly influenced for online HISB by perceived expectations held by people in their social environment, while higher intentions among people perceiving themselves as ill are more strongly influenced by the perceived behavior of relevant others. This can be traced back to the fact that affected individuals observe others as role models in a challenging situation; adopting information-seeking behaviors they perceive others to apply in similar situations may provide a solution for the individual search for help, while the perceived pressure from others appears less important in this situation. The context-dependent role of self-efficacy shows that online HISB intention is only directly affected by self-efficacy for people facing health threats that might result from a higher pressure to search for health-related information in the case of illness. For healthy people, the perception of being capable to gather information appears less relevant as their search for health information lacks urgency. However, self-efficacy has a stronger effect on positive attitudes toward seeking information online. This might indicate that healthy individuals assume that they can benefit from the search for information if and when the corresponding needs arise [66]. Another difference between individuals in contexts of health versus illness was found regarding the influence of perceived knowledge insufficiency on the intention for online HISB. This influence is only significant in the group of ill people, which indicates that sufficient knowledge is particularly perceived relevant when problems occur and information might help; so far, this path has not been confirmed in many studies [14,50]. Besides, the influence of attitudes toward seeking health information on perceived knowledge insufficiency was only apparent for the group of people facing health threats. This suggests that health threats are raising awareness of knowledge insufficiency [39]. Overall, the results suggest that the relative importance of predictors of online HISB differs depending on the individual’s health status. Therefore, considering health status is a valuable extension of theory-based explanatory modeling of online HISB.

### Limitations and Resulting Tasks for Future Research

Although this study informs about the necessity to adjust theoretical models for online HISB, the limitations of the study need to be considered. First, it should be mentioned that information seeking rarely involves only one channel [19,28,29]. Future studies should therefore map the influences of other sources on selecting the internet for health information and compare individual selection factors for different channels. A second limitation is that our study is based on cross-sectional data; therefore, no causality statements can be derived. To ensure a deeper understanding of the processes of information search, longitudinal designs are required in future research. Furthermore, there is a need for cross-cultural studies because the use of the internet to search for health-related topics varies between cultures and countries. We already know that people in the United States are overall more likely to use the internet for health purposes than Europeans [1,2,67]. This suggests that HISB should be systematically compared in terms of cultural context. Third, it should be critically reflected that the categorization of respondents as healthy or ill is unspecific and only based on self-perceptions. Owing to the comparatively small number of cases, differences between acute and chronic illness could not be considered, and different degrees of severity and courses of diseases could not be mapped separately. It can be assumed that, depending on different diseases and different indicators of being or feeling more or less healthy or ill, intentions of HISB will vary [39,66,68]. It should also be noted that the health status variable used to compare the model between contexts of health and illness is closely linked to risk perception as a component of the model. The state of health represents the actual state and can be understood as a predictor of risk perception directed toward the future. Instead of comparing groups of people with different health statuses, further research should integrate health status as a predictor influencing risk perception. Another limitation is associated with the not sufficiently complex measurement of intention to seek information online, as there are manifold types of message forms and contents that can be accessed online (eg, dedicated websites, social media, streaming services, video platforms with user-generated content). Future research should thus consider the diversity to use the internet. Likewise, injunctive and descriptive norms were measured as single items, which should be improved in follow-up studies as well.

### Conceptual Perspectives: Advancing Theorizing on HISB

To sum up, our results indicate that attitudes toward seeking health information online and risk perceptions are of central importance for online HISB across different health statuses. The importance of social norms is generally low for online HISB. Further, predictors such as self-efficacy and perceived knowledge insufficiency play a context-dependent role—they are more influential when individuals are facing health threats and the search for health information is of higher personal relevance and urgency. These findings can be understood as the first step to develop a generalized theory of online HISB. In general, the findings from invariance tests indicate that some of the applied measurements such as risk perception may have limited equivalence in health and illness contexts. This points to limited generalizability of the PRISM, which seems to reach greater explanatory power for ill people who are facing more immediate information needs than for healthy people whose inclination to seek and acquire health-related information is not that much energized by situational circumstances [33,46]. This conceptual challenge also holds implications for measurement and testing the PRISM. Although items should be used that are independent of specific health status, the common way of measuring appears not to be optimal for application across different groups with different health statuses. Thus, further studies are needed to show the extent to which the differences found are valid for different health statuses and different information sources to advance model development and increase understanding of the processes of HISB in more detail.

### Application Perspectives

Effective disseminating of health information in the online domain can benefit from these findings, as the obtained importance of predictors in health and illness conditions allows to characterize target audiences with greater precision. One striking example of such possible benefits is the observation that internet-related self-efficacy makes a substantially greater difference for online HISB among the ill than among the healthy respondents. Providers of information on a specific illness should thus be aware that online platforms may fail to reach out to relevant parts of the target group who are affected by the illness, as only those with higher self-efficacy are likely to access the online information. Additional (channel) strategies beyond online services are thus advised to avoid information underserving of those patients who do not believe in their ability to find and acquire online health information. In contrast, providers of general or prevention-related health information should consider that motivation of healthy individuals to access their content online is primarily influenced by a positive attitude. Thus, although internet resources have low access barriers and are (seemingly) easy to find and acquire, population segments who do not find online HISB desirable are unlikely to make use of the offered information. Hence, among healthy people, the availability, importance, and usefulness of preventive health information on the internet need to be precisely triggered to increase information pull; besides, general health information should still be transported through mass communication channels such as billboards or television that reach out to nonsearching audiences even in times of nearly permanent internet availability and use.

## Appendix

Multimedia Appendix 1 Supplementary data.

## Abbreviations

CFI: comparative fit index
HISB: health information seeking behavior
PRISM: Planned Risk Information Seeking Model
RISP: Risk Information Seeking and Processing Model
RMSEA: root mean square error of approximation
RQ: research question
SRMR: standardized root mean square residual
