# Supplementary material for: Explaining Online Information Seeking Behaviors in People With Different Health Statuses: German Representative Cross-sectional Survey
Source: J Med Internet Res. 2021 Dec 10;23(12):e25963. doi: 10.2196/25963 (PMC8709915; doi:10.2196/25963)
Supplement: Multimedia Appendix 1 [file jmir_v23i12e25963_app1.docx]

## Multimedia Appendix 1. Supplementary data.

### Overview of the Sample Characteristics

**Table S1.** Overview of the sample characteristics of healthy and ill individuals

|  | Healthy individuals  (n=564) | Ill individuals  (n=229) |
| --- | --- | --- |
| Female gender | 55% | 59% |
| Mean age | 43.83 (15.85) | 53.84 (14.92) |
| Education | Less than secondary school leaving certificate: 27.1%  Secondary school leaving certificate: 44.9%  Graduated from high school: 18.1%  University degree: 9.9% | Less than secondary school leaving certificate: 31.5%  Secondary school leaving certificate: 46.3%  Graduated from high school: 8.7%  University degree: 13.5% |

### Attitude Toward Seeking Online

The measurement model fits the data fairly well (χ² (14)=51.515, *P*≤.01; CFI=0.981; RMSEA=0.057; SRMR=0.018, Cronbach α=.944), and all factor loadings were ≥ .80. To compare the groups of people perceiving themselves as ill or healthy, we assessed measurement invariance. The results showed satisfying equivalence (see Table S2).

Table S2. Measurement invariance of attitude toward seeking online

| Model | Fit to the Data | | | | Model comparison | | | |
| --- | --- | --- | --- | --- | --- | --- | --- | --- |
|  | *Χ^2^* | Df | CFI | RMSEA | Δ *Χ^2^* | Δ df | Δ *P* | Δ CFI |
| Configural Invariance | 157.96 | 28 | 0.972 | 0.074 | - | - | - | - |
| Metric  Invariance | 176.49 | 34 | 0.967 | 0.073 | 16.99 | 6 | .009 | 0.005 |
| Scalar  Invariance | 186.48 | 40 | 0.964 | 0.071 | 10.25 | 6 | .114 | 0.003 |

n=793

### Information-Related Self-Efficacy

The data fit of the measurement model as well as the factor loading (≥ .71) were satisfying (χ² (20)=65.044, *P*≤.01; CFI=0.983; RMSEA=0.052; SRMR=0.020; Cronbach α=.947). The assessment of measurement invariance showed high equivalence for the measurement in both groups. The structure (configurable invariance) and the factor loading (metric invariance) as well as the regression constants (scalar invariance) were considered comparable (see Table S3).

Table S3. Measurement invariance of information-related self-efficacy

| Model | Fit to the Data | | | | Model Comparison | | | |
| --- | --- | --- | --- | --- | --- | --- | --- | --- |
|  | *Χ^2^* | Df | CFI | RMSEA | Δ *Χ^2^* | Δ df | Δ *P* | Δ CFI |
| Configural Invariance | 164.85 | 40 | 0.980 | 0.057 | - | - | - | - |
| Metric  Invariance | 170.56 | 47 | 0.980 | 0.054 | 5.29 | 7 | .625 | 0.001 |
| Scalar  Invariance | 176.05 | 54 | 0.979 | 0.051 | 5.75 | 7 | .567 | 0.001 |

n=793

### Risk Perception

The model fit was excellent (χ² (1)=0.281, n.s.; CFI=1.000; RMSEA=0.000; SRMR=0.007; Cronbach α=.863), but the measurement invariance in the comparison of people perceiving themselves as healthy or ill indicated that the equivalence of the measurement had to be evaluated as critically (see Table S4). This can be attributed to high but different factor loadings in both groups. Therefore, it can be assumed that there are differences in the understanding of the measurement model between the two groups, and the adequacy of the measurement for both healthy and ill persons must be questioned. However, since high factor loadings were achieved for both groups, inclusion in the group comparison is viewed as justified.

Table S4. Measurement invariance of risk perception.

| Model | Fit to the Data | | | | Model Comparison | | | |
| --- | --- | --- | --- | --- | --- | --- | --- | --- |
|  | *Χ^2^* | Df | CFI | RMSEA | Δ *Χ^2^* | Δ df | Δ *P* | Δ CFI |
| Configural Invariance | 15.42 | 2 | 0.975 | 0.100 | - | - | - | - |
| Metric Invariance | 19.88 | 3 | 0.966 | 0.096 | 3.77 | 1 | .052 | 0.009 |
| Scalar  Invariance | 35.75 | 5 | 0.931 | 0.106 | 14.07 | 2 | .000 | 0.035 |

n=793

### Affective Response to Risks

The fit of the measurement model can be rated as good to very good (χ² (1)=2.806, n.s.; CFI=0.996; RMSEA=0.047; SRMR=0.016; Cronbach α=.963). Analogous to risk perceptions, there were problems with the equivalence of measurement in the two groups of people perceiving themselves as healthy or ill (see Table S5), which must be considered when interpreting the results.

**Table S5.** Measurement invariance of affective response to risks.

| Model | Fit to the Data | | | | | Model Comparison | | | | |
| --- | --- | --- | --- | --- | --- | --- | --- | --- | --- | --- |
|  | *Χ^2^* | Df | CFI | RMSEA | Δ *Χ^2^* | | Δ df | Δ *P* | Δ CFI |  |
| Configural Invariance | 13.29 | 2 | 0.981 | 0.099 | - | | - | - | - |  |
| Metric Invariance | 14.94 | 3 | 0.979 | 0.083 | 1.30 | | 1 | .254 | 0.001 |  |
| Scalar  Invariance | 35.07 | 5 | 0.934 | 0.115 | 25.07 | | 2 | .000 | 0.045 |  |

n=793

### Intention to seek information online

The measurement model fit the data very well (χ² (1)=0.294, n.s.; CFI=1.000; RMSEA=0.000; SRMR=0.004; Cronbach α=.920), the single items showed high factor loadings (≥ .81), and the measurement invariance confirmed the equivalence of the instrument (see Table S6).

Table S6. Measurement invariance of intention to seek information online.

| Model | Fit to the Data | | | | Model comparison | | | |
| --- | --- | --- | --- | --- | --- | --- | --- | --- |
|  | *Χ^2^* | Df | CFI | RMSEA | Δ *Χ^2^* | Δ df | Δ *P* | Δ CFI |
| Configural Invariance | 2.023 | 2 | 1.000 | 0.017 | - | - | - | - |
| Metric Invariance | 2.083 | 3 | 1.000 | 0.000 | .04 | 1 | .829 | 0.000 |
| Scalar  Invariance | 4.153 | 5 | 1.000 | 0.000 | 2.13 | 2 | .345 | 0.000 |

n=793
